# Supplementary material for: Systematic pharmacological screens uncover novel pathways involved in cerebral cavernous malformations
Source: EMBO Mol Med. 2018 Sep 4;10(10):e9155. doi: 10.15252/emmm.201809155 (PMC6180302; doi:10.15252/emmm.201809155)
Supplement: Supplementary file 1 — Expanded View Figures PDF [file EMMM-10-e9155-s001.pdf]

Expanded View Figures

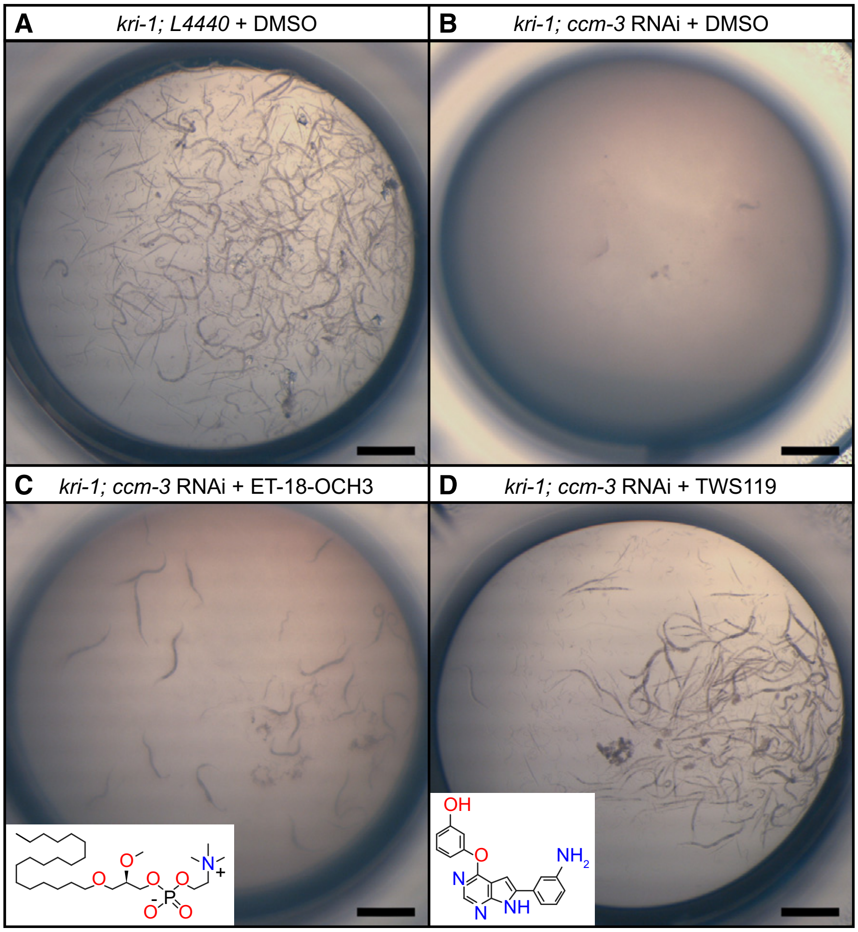

Figure EV1. Small-molecule drug screen in *C. elegans* identifies compounds relevant for CCM.

A *kri-1* mutant worms treated with control L4440 RNAi and with DMSO are viable. Shown is a representative picture of a control well from a 96-well plate.

B Treatment of *kri-1* mutants with *ccm-3* RNAi causes synthetic lethality. Incubation of this strain with DMSO (control) has no further effect on the synthetic lethality.

C Incubation of the *ccm-3* RNAi-treated *kri-1* mutants with the phospholipase C inhibitor ET-18-OCH3 results in a mild rescue, as seen by the presence of a few worms.

D Incubation of the *ccm-3* RNAi-treated *kri-1* mutants with the GSK-3/PI3K/Akt/mTOR inhibitor TWS119 strongly rescues synthetic as indicated by the high number of worms.

Data information: All scale bars are 1 mm.

 © 2018 The Authors

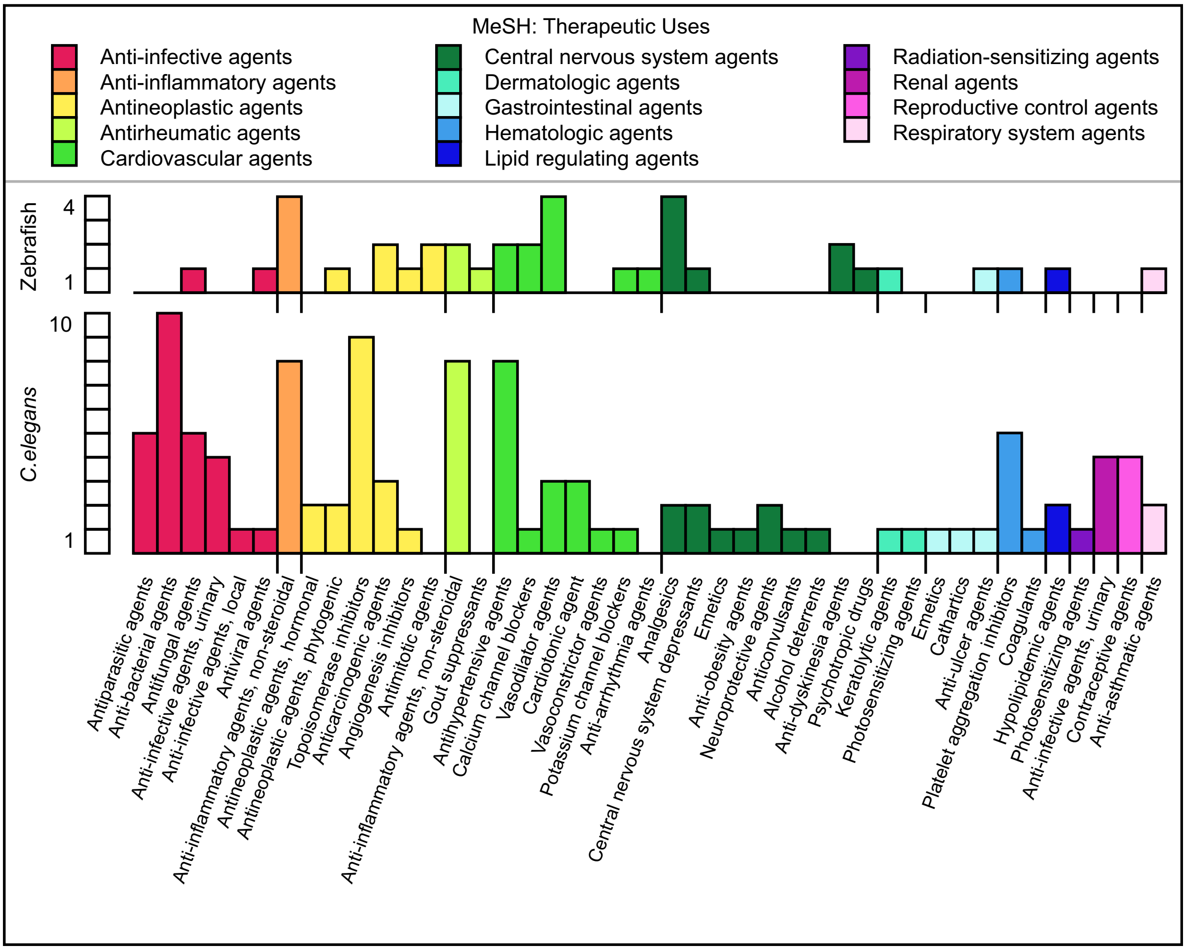

Figure EV2. Representation of MeSH terms for therapeutic uses obtained for some of the active compounds identified in the zebrafish and *C. elegans* screens.

Figure EV3. Representation of MeSH terms obtained for some of the active compounds identified in the zebrafish and *C. elegans* screens.

A MeSH terms for physiological effects.
B MeSH terms for molecular mechanisms.

▶

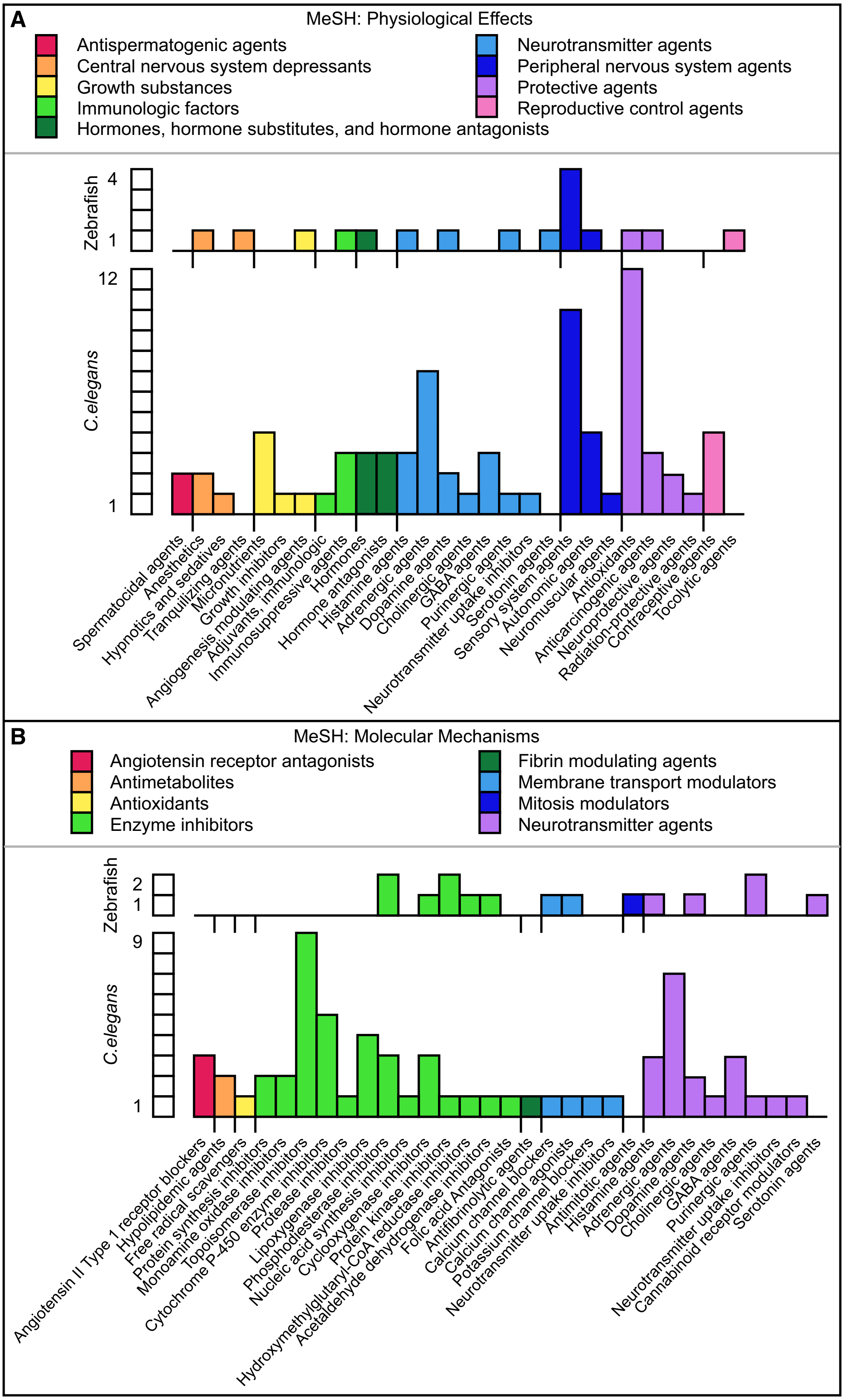

Figure EV3.

© 2018 The Authors

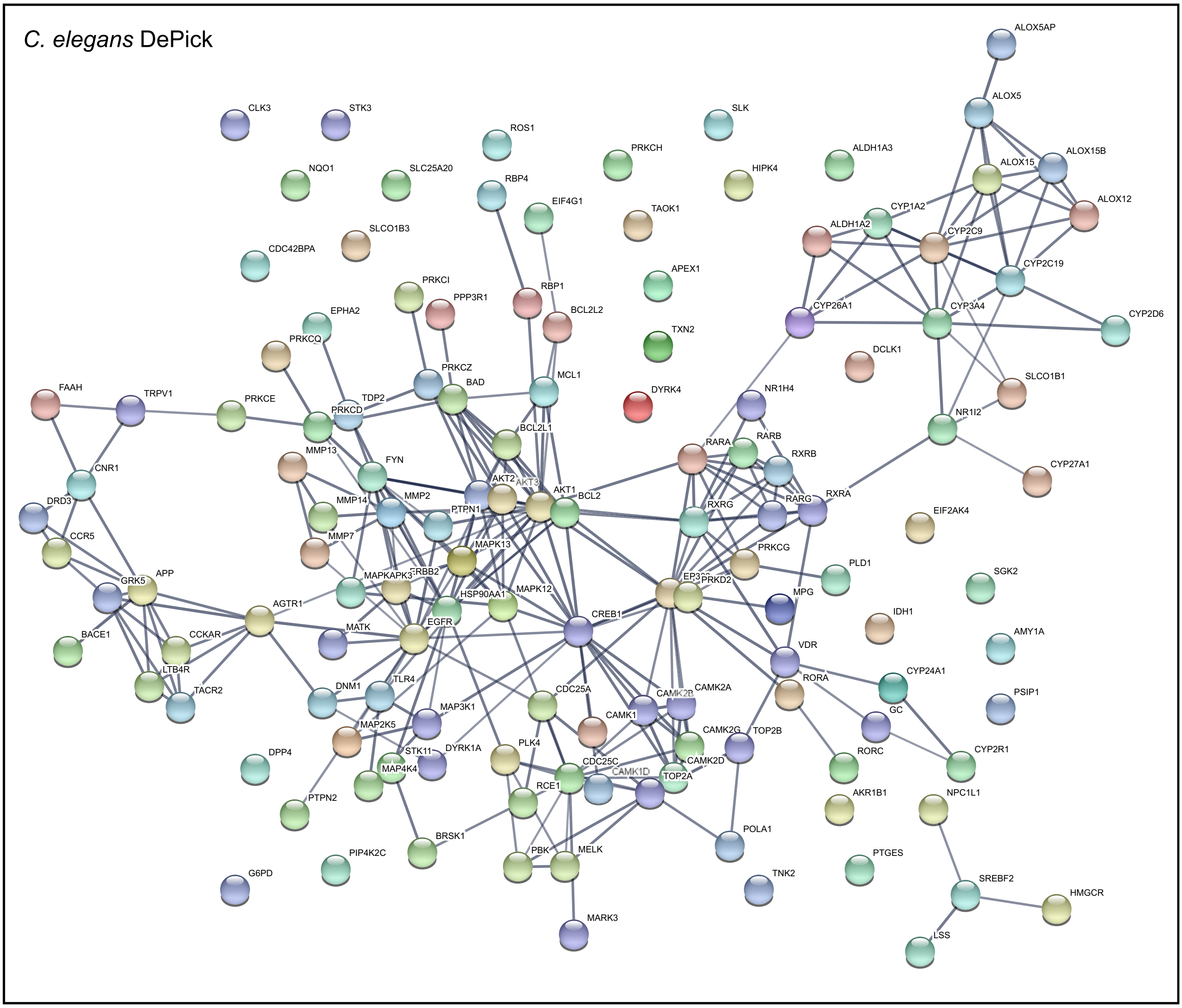

Figure EV4. STRING network clustering for interactions of *C. elegans* DePick target proteins.

Nodes are DePick target proteins of compounds identified in the *C. elegans* screen. Edges are protein–protein associations. Line thickness represents the strength of data support.

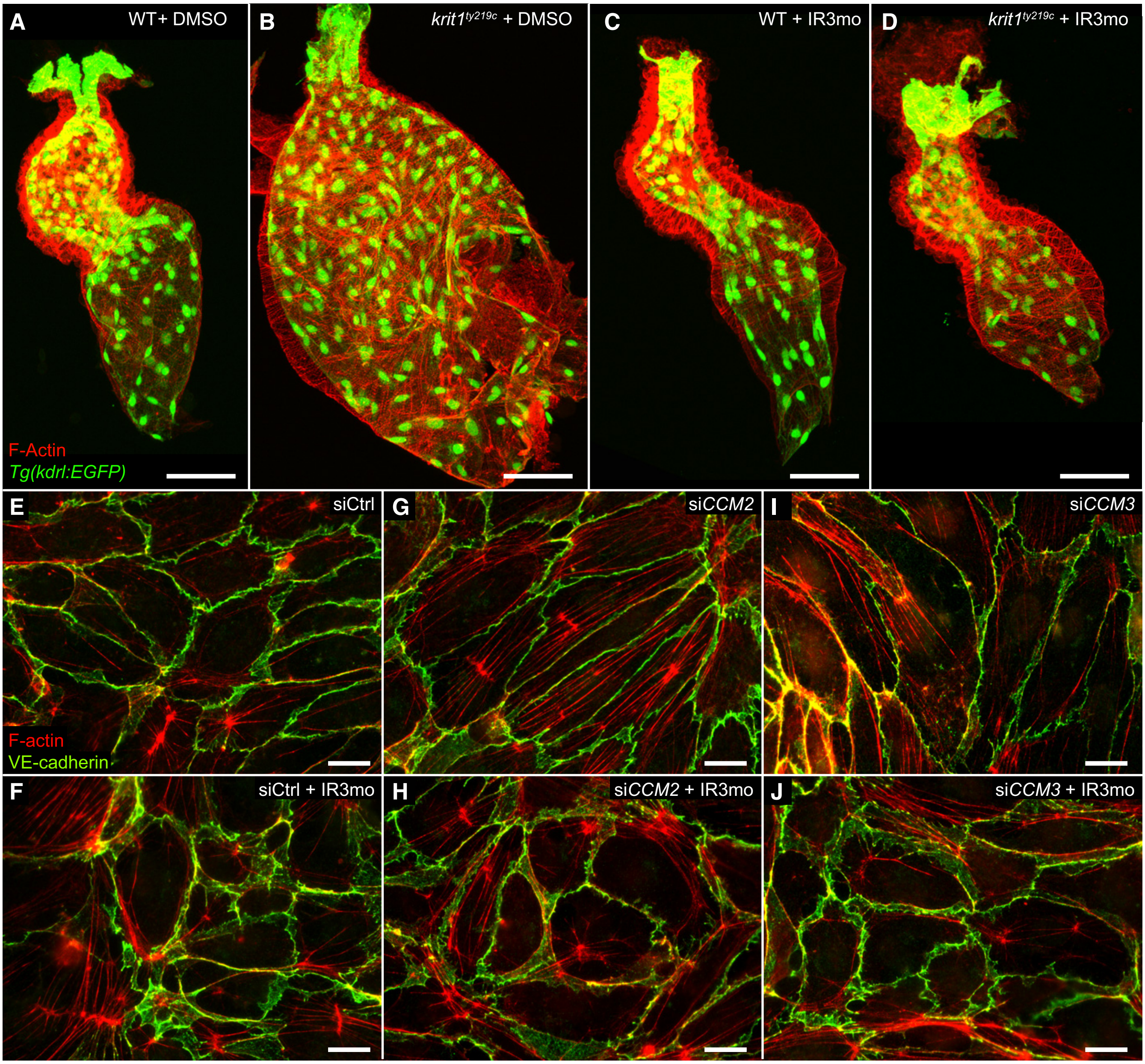

Figure EV5. Indirubin-3-monoxime (IR3mo) rescues the CCM phenotype in *krit1*^{ty219c} mutant zebrafish and CCM2- and CCM3-depleted HUVECs.

A–D Treatment with 5 μM IR3mo rescues the embryonic zebrafish *krit1*^{ty219c} mutant ballooning heart phenotype. Shown are images of confocal *z*-scan projections of wild-type (WT) (A, C) and *krit1*^{ty219} mutant (B, D) zebrafish embryonic hearts at 48 hpf expressing the endothelial reporter *Tg*(*kdrl:GFP*)^{s843} (green) and counter-stained for ACTIN (red). Scale bar is 50 μm.

E–J Treatment with 10 μM IR3mo for 48 h rescued the CCM phenotype in CCM2- or CCM3-depleted HUVECs. Shown are confocal images of HUVECs with labeled VE-cadherin (green) and F-ACTIN (red). Control siRNA-silenced (E, F), *CCM2* siRNA-silenced (G, H), or *CCM3* siRNA-silenced (I, J) HUVECs untreated (E, G, I) or treated (F, H, J) with IR3mo. Scale bar is 10 μm.

 © 2018 The Authors
