# Supplementary figures and images for: Systematic pharmacological screens uncover novel pathways involved in cerebral cavernous malformations
Source: EMBO Mol Med. 2018 Sep 4;10(10):e9155. doi: 10.15252/emmm.201809155 (PMC6180302; doi:10.15252/emmm.201809155)

Fig4D

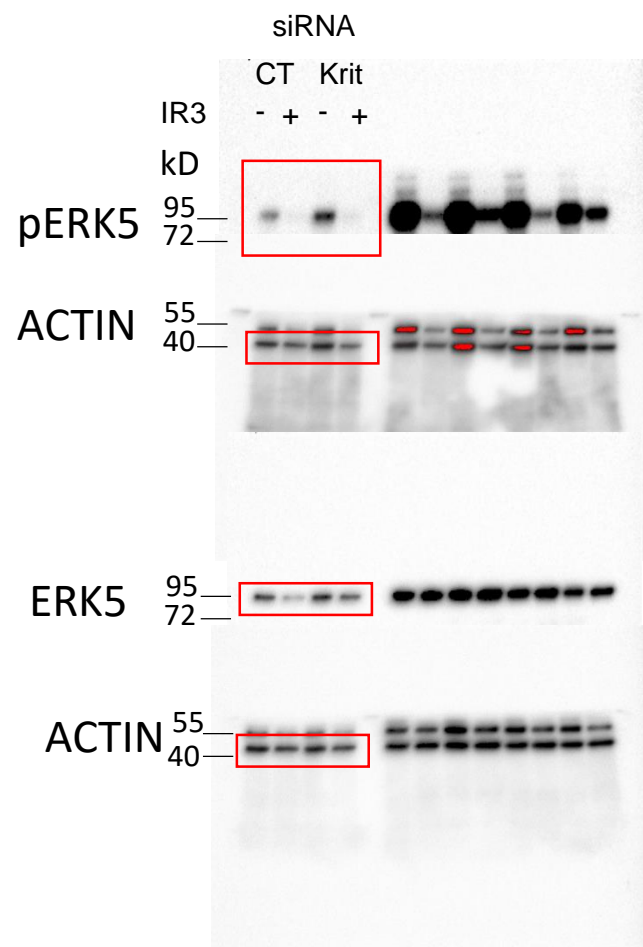

Supplement: Supplementary file 13 — Source Data for Figure 4 [file EMMM-10-e9155-s012.pdf]
